# Supplementary material for: User-Friendly Reverse Genetics System for Modification of the Right End of Fowl Adenovirus 4 Genome
Source: Viruses. 2020 Mar 11;12(3):301. doi: 10.3390/v12030301 (PMC7150739; doi:10.3390/v12030301)
Supplement: Supplementary file 1 [file viruses-12-00301-s001.pdf]

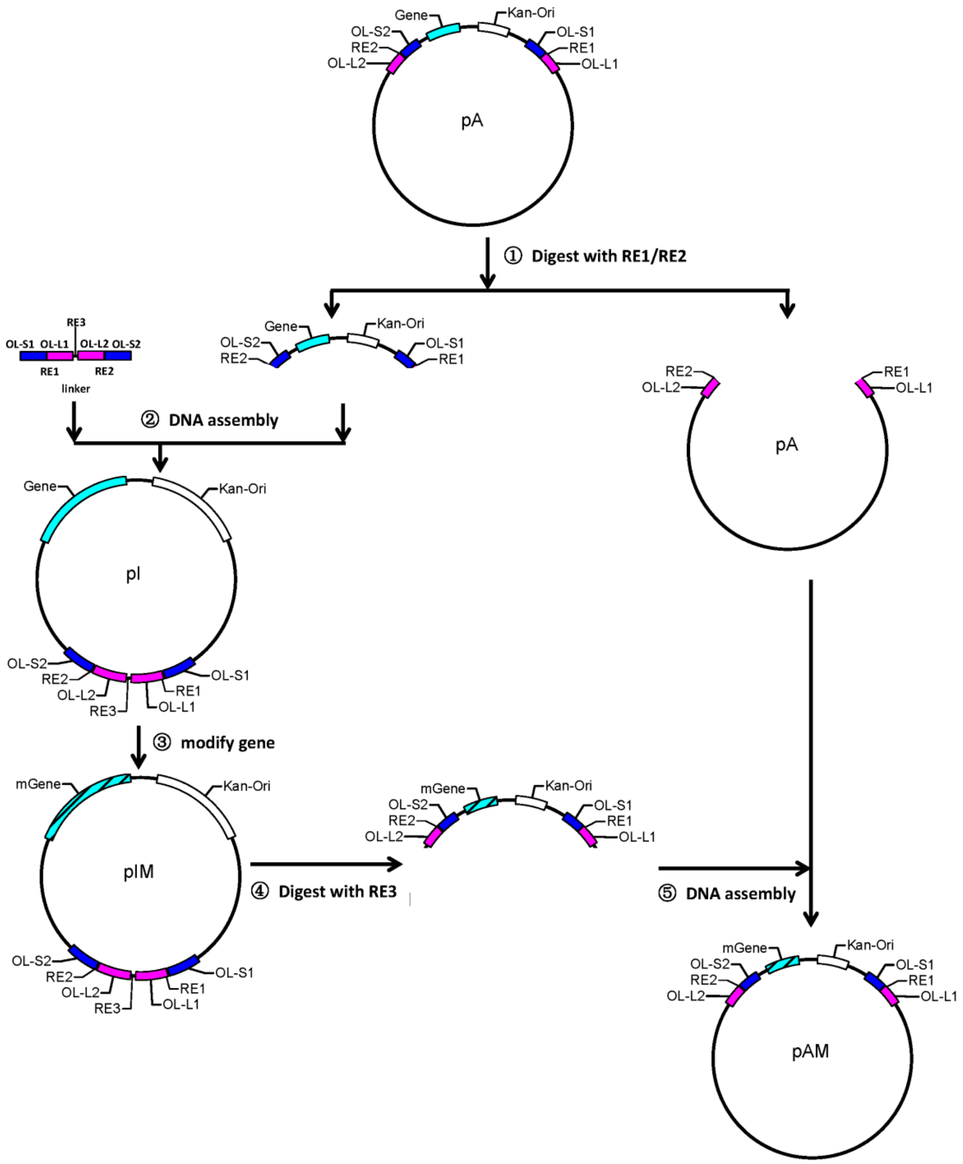

Figure S1. Schematic diagram to show the principle of adenoviral reverse genetics system working through restriction-assembly. ① Sequence of adenoviral infectious clone plasmid (pA) is analyzed to find two unique cutter Restriction Enzymes (RE, when RE1≠RE2) or one dual cutter (when RE1=RE2), the plasmid pA is digested with RE1/RE2, and the small fragment carrying target gene (Gene) and plasmid backbone (Kan-Ori) is recovered. ② a linker containing sequences flanking RE1 and RE2 and another restriction enzyme site (RE3) is synthesized directly or generated by extension of two annealed single-stranded DNA oligos. The short sequences flanking RE1 and RE2 (OL-S1, S2, L1 and L2) are OverLaps with a length of 15-30 bp required for generating the Small plasmid (intermediate plasmid, pI) or Large plasmid (modified adenoviral plasmid, pAM) through DNA assembly. RE3 should be an 8-bp cutter and does not digest the small fragment exised from pA with RE1/RE2. The intermediate plasmid (pI) is generated by fusing the linker and the small fragment of pA through DNA assembly. ③ Gene in pI is modified with the method of overlap extension PCR-mediated site-directed mutation to generate pIM (modified intermediate plasmid). ④⑤ pIM is linearized with RE3 and fused to the large fragment from pA to generate the modified adenoviral plasmid (pAM) through DNA assembly. pAM is now ready for recombinant virus rescue.

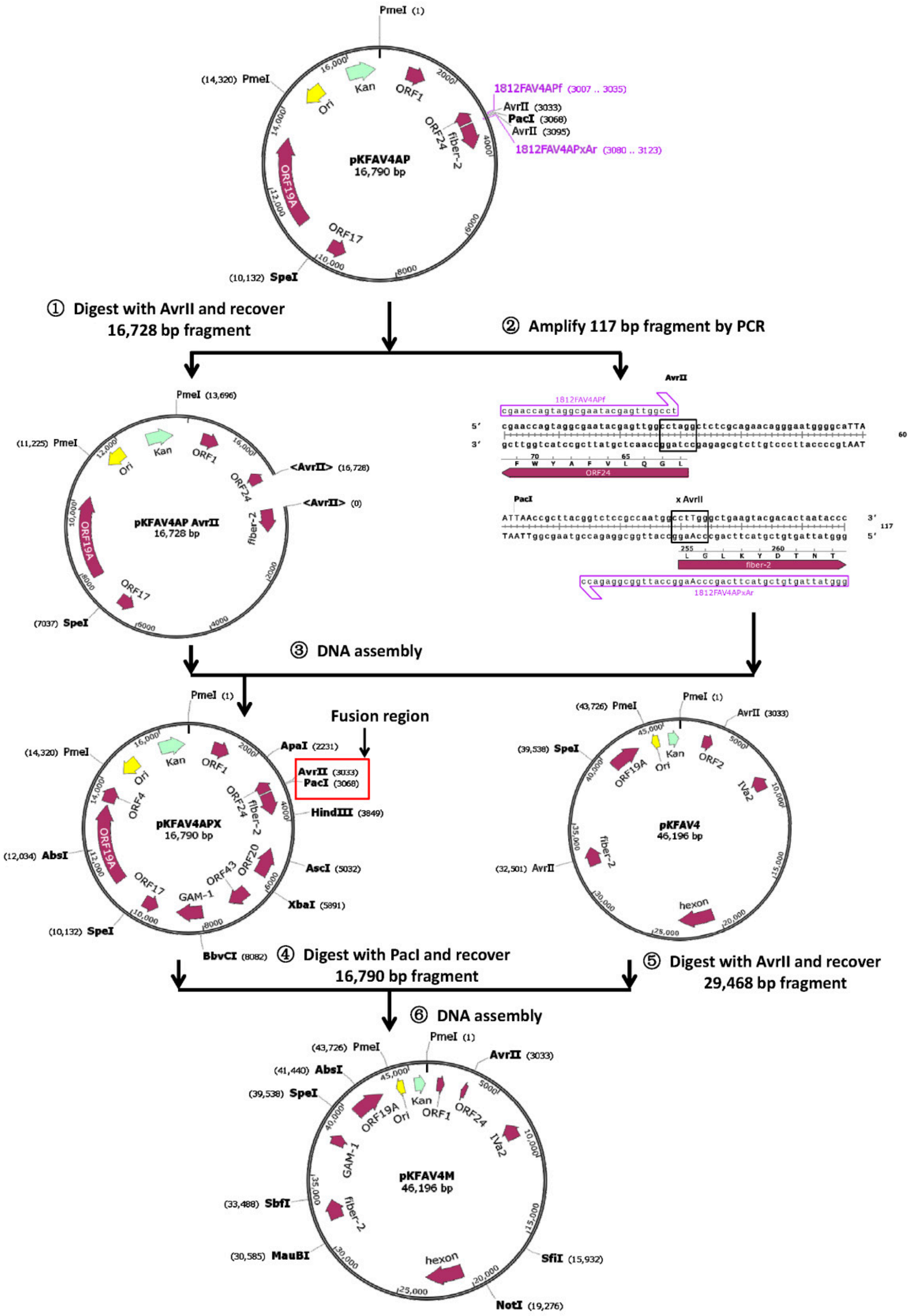

Figure S2. Construction of a modified infectious clone of FAdV-4 (pKFAV4M, pKFAV4 with Mutated AvrII site), in which the AvrII site in fiber2 gene was removed without change of the encoded amino acid.

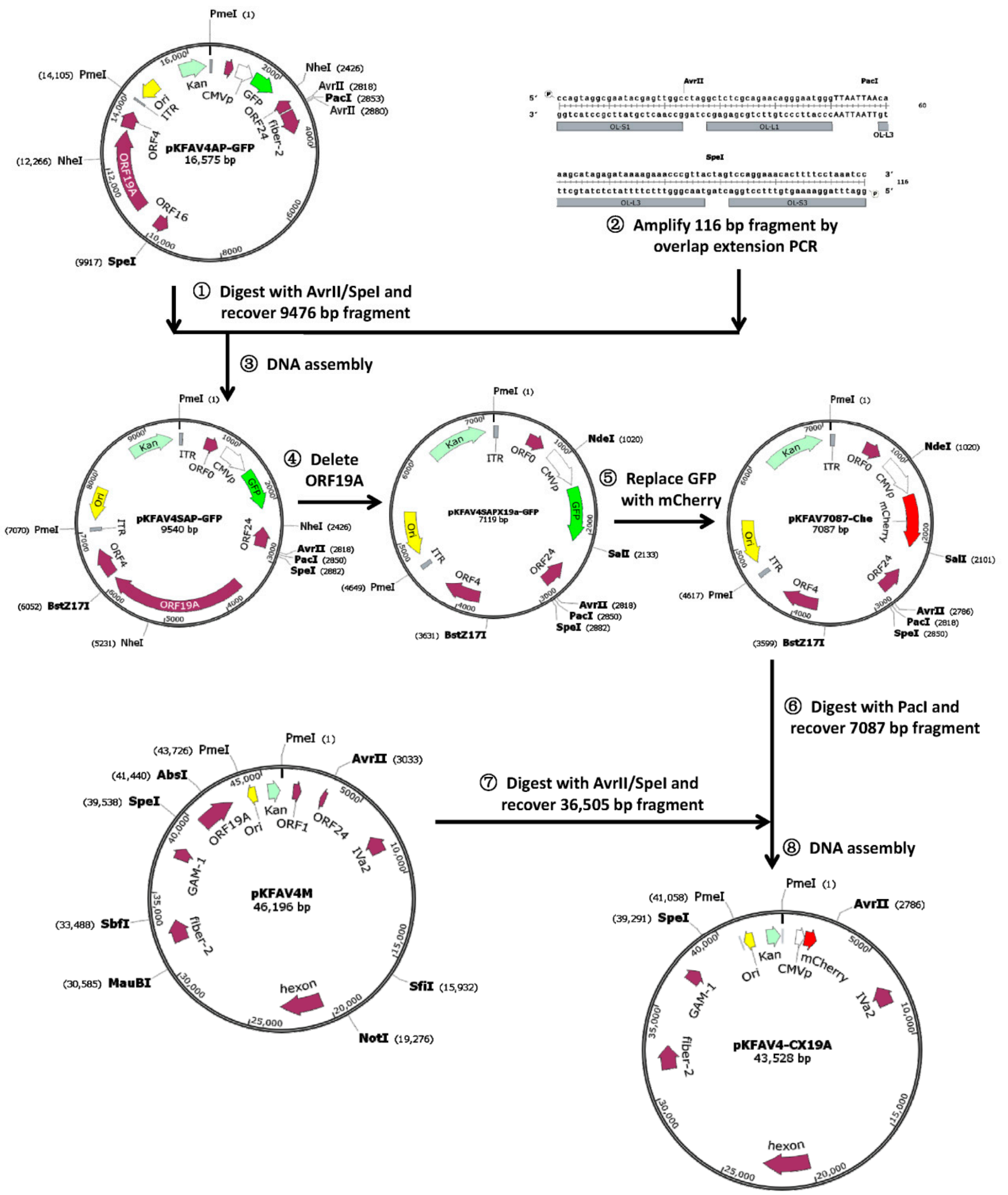

Figure S3. Construction of ORF19A-deleted and mCherry-carrying adenoviral plasmid pKFAV4-CX19A. A smaller intermediate plasmid pKFAV4SAP-GFP was generated, which could work together with pKFAV4M as a reverse genetics system to modify the genomic sequence downstream of the SpeI site on the right end. The ORF19A CDS was deleted and the CDS of GFP was replaced with that of mCherry in pKFAV4SAP-GFP to generate pKFAV7087-Che intermediate plasmid. pKFAV7087-Che was linearized with PacI and fused to the large fragment of SpeI/AvrII-digested pKFAV4M to generate adenoviral plasmid pKFAV4-CX19A by DNA assembly.

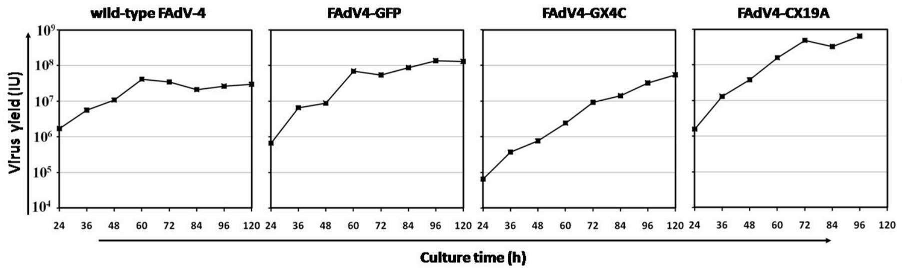

Figure S4. One-step growth curve of wild-type and recombinant FAdV-4 viruses in LMH cells. LMH cells were seeded in 12-well plates, infected with wild-type FAdV-4 at an MOI of 200 vp/cell or recombinant viruses at an MOI of 30 vp/cell for 2 hours. Cells in each well were maintained in 1 ml DMEM plus 2% FBS in the following 5 days without medium change. Cells and culture supernatants were collected at the indicated time points post infection. Viruses were titrated on LMH cells with the limiting dilution method. For wild-type FAdV-4 titration, immunocytochemistry was performed to find the virus-replicating cells with sera from chickens that survived FAdV-4 infection as the primary antibody [13]. For recombinant FAdV-4 titration, GFP+ or mCherry+ cells were counted under fluorescence microscope directly as described in the Materials and methods section. The yields of progeny viruses in each well were calculated and used to draw the growth curves.

[13] Zou, X.H.; Bi, Z.X.; Guo, X.J.; Zhang, Z.; Zhao, Y.; Wang, M.; Zhu, Y.L.; Jie, H.Y.; Yu, Y.; Hung, T., et al. DNA assembly technique simplifies the construction of infectious clone of fowl adenovirus. J Virol Methods 2018, 257, 85-92, doi:10.1016/j.jviromet.2018.04.001.
